# Recurrent brain metastases: the role of resection of in a comprehensive multidisciplinary treatment setting

**DOI:** 10.1186/s12885-022-09317-6

**Published:** 2022-03-15

**Authors:** Nadine Heßler, Stephanie T. Jünger, Anna-Katharina Meissner, Martin Kocher, Roland Goldbrunner, Stefan Grau

**Affiliations:** 1 Center for Neurosurgery, Department of General Neurosurgery, Faculty of Medicine, University Hospital Cologne, University of Cologne, Cologne, Germany; 2 Centre for Integrated Oncology, Faculty of Medicine and University Hospital Cologne, University of Cologne, Cologne, Germany; 3 Center for Neurosurgery, Department of Stereotactic and Functional Neurosurgery, Faculty of Medicine and University Hospital Cologne, University of Cologne, Cologne, Germany; 4 Department of Neurosurgery, Klinikum Fulda gAG, Academic Hospital of the University of Marburg, Fulda, Germany

**Keywords:** Recurrent brain metastasis, Radio-oncological treatment, Overall survival

## Abstract

**Background:**

Treatment decision for recurrent symptomatic brain metastases (BM) is challenging with scarce data regarding surgical resection. We therefore evaluated the efficacy of surgery for pretreated, recurrent BM in a comprehensive multidisciplinary treatment setting.

**Methods:**

In a retrospective single center study, patients were analyzed, who underwent surgical resection of recurrent BM between 2007 and 2019. Intracranial event-free survival (EFS) and overall survival (OS) were evaluated by Kaplan-Maier and Cox regression analysis.

**Results:**

We included 107 patients with different primary tumor entities and individual previous treatment for BM. Primary tumors comprised non-small cell lung cancer (NSCLC) (37.4%), breast cancer (19.6%), melanoma (13.1%), gastro-intestinal cancer (10.3%) and other, rare entities (19.6%). The number of previous treatments of BM ranged from one to four; the adjuvant treatment modalities comprised: none, focal or whole brain radiotherapy, brachytherapy and radiosurgery. The median pre-operative Karnofsky Performance Score (KPS) was 70% (range 40–100) and improved to 80% (range 0-100) after surgery. The complication rate was 26.2% and two patients died during the perioperative period. Sixty-seven (62.6%) patients received postoperative local radio-oncologic and/or systemic therapy. Median postoperative EFS and OS were 7.1 (95%CI 5.8–8.2) and 11.1 (95%CI 8.4–13.6) months, respectively. The clinical status (postoperative KPS ≥ 70 (HR 0.27 95%CI 0.16–0.46; *p* < 0.001) remained the only independent factor for survival in multivariate analysis.

**Conclusions:**

Surgical resection of recurrent BM may improve the clinical status and thus OS but is associated with a high complication rate; therefore a very careful patient selection is crucial.

## Background

Due to rising medical standards, multidisciplinary treatment options including novel therapeutic regimens, the number of patients with brain metastases (BM) is increasing [1–4]. Although BM are considered, in principle, a fatal event for oncological patients, treatment paradigms are changing, and affected patients are nowadays frequently treated with repeated non-invasive therapeutic procedures such as radiotherapy and systemic oncological treatments. While the role of neurosurgical resection of primary and symptomatic BM is clearly defined [5, 6], the application of surgery for recurrent BM, especially after previous multimodal treatments, remains an individual decision [7], particularly since underlying studies [8, 9] are scarce and mostly focus on narrowly defined, rather than heterogeneously pretreated “real-life” patient cohorts.

In the light of an increasing number of multidisciplinary comprehensive oncological treatmentoptions, including several types of focused radiotherapy and targeted medical treatments with a reported overall survival (OS) benefit, the role of neurosurgery in the context of relapse, especially for symptomatic BM, needs to be clearly defined.

## Methods

### Selection of study population

For this retrospective, monocentric cohort study, we queried our database for patients who had undergone resection of previously treated, large recurrent BM in our department between 2007 and 2019 and in whom a recurrence was confirmed by histopathology. The following parameters were identified: demographic/baseline characteristics (gender, age at time of diagnosis and at time of surgery of the recurrent BM), tumor characteristics (type of primary tumor, local and systemic tumor status, number and location of recurrent BM, time to recurrence since initial cancer diagnosis, time to recurrence since initial diagnosis of BM), therapeutic interventions (previous treatment, types of adjuvant therapy, number of previous recurrences), clinical status (neurological symptoms, pre- and postoperative Karnofsky-Performance-Scale (KPS)), and outcome measures (surgery-related complications, time to further recurrence after surgery). Data were retrieved from the electronic hospital database and paper charts. The study was approved by the local ethical committee (reference number: 18–089).

### Indication for surgery

Recurrent BM was diagnosed by magnetic resonance imaging (MRI) or, if required, amino acid positron emission tomography (PET). All treatment decisions were made within an interdisciplinary institutional tumor board comprising board-certified neurosurgeons, neuro-oncologists, medical oncologists, neuro-radiologists, neuropathologists, and palliative care physicians. In general, criteria for (re-)operation were large tumors, symptomatic brain edema, safe accessibility of the lesion allowing safe resection, a fair clinical condition or BM-associated symptoms, adjuvant treatment options (re-irradiation, chemotherapy, or molecular therapy), necessity for obtaining tissue diagnosis, rapid progression leading to neurological complications, or no less invasive treatment options other than surgery. Histopathological diagnosis was made by the local Departments of Neuropathology or Pathology.

### Surgical treatment and follow-up

The extent of resection was assessed by early postoperative MRI performed within 48 h after surgery and classified as gross total resection when no residual contrast-enhancing tissue was visible on T1-weighted imaging. Any residual contrast enhancement was defined as subtotal resection. Clinical and radiological follow-up was performed in three-monthly intervals. Intracranial failure was defined as newly developing contrast enhancement in brain MR imaging.

Complications were classified according to the Common Terminology Criteria of Adverse Events (CTCAE) by the National Cancer Institute (NCI) [10, 11].

### Statistical analysis

For descriptive statistics, continuous values are given in median and range, ordinal and categorical variables are stated in numbers and percentages. Post-surgical survival time was calculated from the date of surgery to date of death or last follow-up; patients alive at the time of their last follow up were censored. Event-free survival (EFS) was assumed in the case of no intracranial relapse. Predictive variables for both endpoints were identified by univariate and multivariate analysis. For categorical variables, the log-rank test was used to identify covariates with an influence on EFS and OS and visualized in Kaplan-Meier plots. For continuous variables, Hazard ratios were calculated using Cox regression. P-values < 0.05 were considered statistically significant. Variables with a significant impact were included in a multivariate Cox regression model. All statistical analyses were performed using SPSS Statistics Version 25 (*IBM, Armonk, NY, USA*).

## Results

### Baseline parameters and demographics

The study included 107 patients with a median age of 61 (range 26–83) years at the time of operation. Forty-three patients (40.2%) were male. Primary tumor entities comprised non-small cell lung cancer (NSCLC) (37.4%), breast cancer (19.6%), melanoma (13.1%), gastro-intestinal tumor (GIT) (10.3%) and other, rare entities (19.6%). At the time of BM relapse, extracranial metastases were present in 61 (57.0%) patients. Detailed demographic and clinical data are displayed in Table 1.

**Table 1:** Complications stratified according to CTCAE (Common Terminology Criteria of Adverse Events)

Complication	CTCAE grade	n
New neurological deficit	2	6
Wound healing disorder	2	5
Wound healing disorder requiring surgery (revision, external drain)	3	4
CSF disorder requiring surgery	3	3
Postoperative haemorrhage requiring intervention	4	1
Cerebral ischemia	4	1
Cerebral edema	4	1
Pulmonary embolism	3	2
Carotic artery dissection	4	1
Pneumonia, sepsis	3	2
Postoperative death	5	2

### Previous treatment and clinical status at time of recurrence

Previous cerebral treatment comprised one or more local and/or systemic therapies including surgery, whole brain radiation therapy (WBRT), focal/partial brain radiation therapy (fRT), stereotactic radiosurgery (sRS) and brachytherapy (BT). The number and detailed information on previous treatment modalities were recorded (Table 2). At the time of resection, 79 (73.8%) patients suffered from BM-related symptoms including vertigo, hemiparesis, cognitive impairment, epilepsy, and headache. The median preoperative Karnofsky performance scale (KPS) was 70 (range 40–100).

**Table 2:** Baseline demographic characteristics and parameters

Parameter	No.	%	Median	Range
Age at operation			61	26–83
≤ 65 years	75	70.1		
> 65 years	32	29.9		
Gender				
Male	43	40.2		
female	64	59.8		
Primary tumor				
non-small cell lung cancer	40	37.4		
Breast cancer	21	19.6		
Melanoma	14	13.1		
Gastro-intestinal tumorOther	11	10.3		
Other	21	19.6		
Extracranial disease				
Stable	46	43.0		
Non-stable	61	57.0		
Symptoms (multiple references possible)				
Cerebellar	19	17.8		
Cognitive	11	10.3		
Hemiparesis	25	23.4		
Seizures	14	13.1		
Headache	22	20.6		
Impaired vision	15	14.0		
Aphasia	13	12.1		
Others	18	16.8		

### Surgical treatment, complications, and adjuvant treatment

At time of surgery 80 (74.8%) patients suffered from a single recurrent BM, 19 patients (17.8%) from oligo- (2–3) BM and eight patients (7.5%) from multiple (≥ 4) BM. Resection of the target lesion was complete (gross total resection) in 78 (72.9%) patients. Surgery was performed in all patients under general anesthesia with the aid of neuro-navigation, ultrasound, and intra-operative monitoring, if required. Surgery improved the Karnofsky performance scale to a median of 80 (0-100). After resection, adjuvant local treatment was administered in 67 patients (62.6%), comprising WBRT (*n* = 5), fRT (*n* = 49), stereotactic radiosurgery (*n* = 11), or a combination of the latter two (*n* = 2). Medical treatment was initiated or continued in 37 (34.6%) patients (Table 3). Surgery-related complications occurred in 28 patients (26.2%) with two patients dying during the acute phase. Details on postsurgical complications and their grading are displayed in detail in Table 3.

**Table 3:** Pre- and postsurgical treatment, surgery, and complications

Parameter	No.	%
**Previous treatment**		
Resection	44	41.1
Radiotherapy		
Whole brain radiotherapy	30	28.0
Partial brain radiotherapy	24	22.4
Stereotactic radiosurgery	53	49.5
Brachytherapy	8	7.5
**Number of recurrent BM**		
1 BM	80	74.8
2–3 BM	19	17.8
≥ 4 BM	8	7.5
**Extent of resection**		
Gross total	78	72.9
Subtotal	29	27.1
**Adjuvant local treatment**		
None	40	37.4
Radiotherapy	67	62.6
Whole brain radiotherapy	5	4.7
Partial brain radiotherapy	49	45.8
Stereotactic radiosurgery	11	10.3
Combination	2	1.9
**Postsurgical systemic therapy**	37	34.6
**Cause of death (***n* = 73)		
Neurological	37	34.6
Systemic	12	11.2
Others	2	1.9
unknown	22	20.6

### Survival

In 51 patients (47.7%), a cerebral treatment failure was detected, resulting in a median EFS of 7.1 (95%CI 5.8–8.2) months. None of the factors analyzed influenced EFS.

At the time of analysis, 73 (68.2%) patients had died. Median OS time was 11.1 (95%CI 8.4–13.6) months. Three patients (2.8%) died within the first 30 days after surgery, two from surgical complications. In the remaining cohort, the causes of death were systemic disease progression in 12 patients (11.2%), cerebral progression in 37 patients (34.6%) and other causes in two patients (1.9%). In the remaining patients, the cause of death was unspecified.

**Fig. 1:**
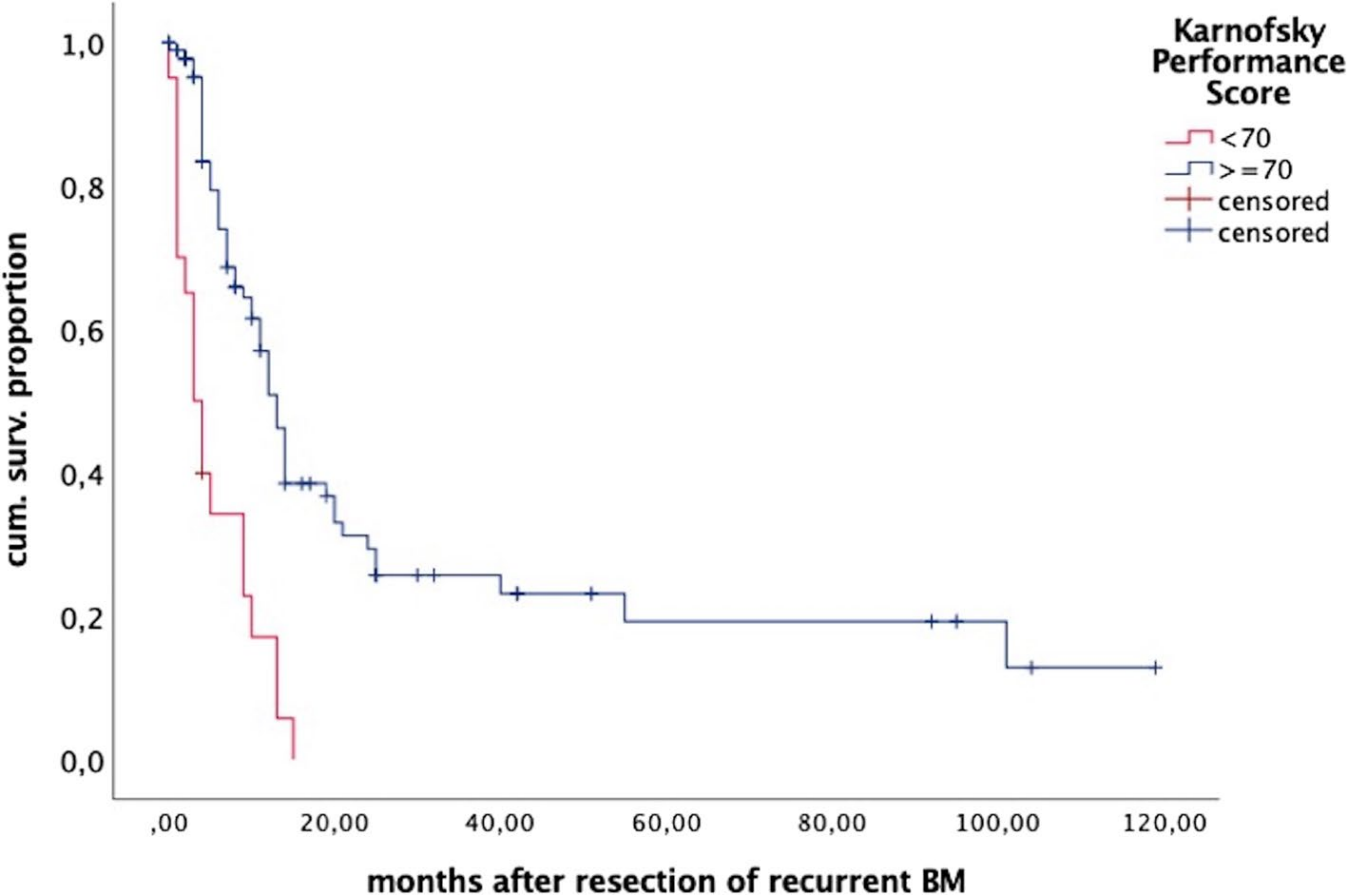
Overall survival (OS), depicting the impact of the clinical status after surgery. Kaplan-Meier plot

In univariate analysis, a pre- and postoperative KPS ≥ 70 (*p* = 0.002 and *p* < 0.001, Fig. 1) and neurological symptoms caused by BM (*p* = 0.036) were prognostic for survival, while all other parameters (age, primary, number of BM, location, previous treatment, application and type of local treatment, ongoing systemic treatment, extracranial status) showed no significant impact. In multivariate analysis only the postoperative clinical status (HR 0.207 95%CI 0.0816–0.3436; *p* < 0.001) remained independent.

## Discussion

Due to closer surveillance during follow-up with routine MR imaging, an increasing number of interdisciplinary treatment options, including effective systemic therapies, the number of patients diagnosed with recurrent BM is increasing [1–4]. However, the inevitable question of how to treat these patients adequately after cerebral progression still remains unsolved, especially for patients maintaining a good clinical condition over a longer period of time before BM recurrence [4]. Most studies with respect to treatment of recurrent BM focus on a single treatment option such as (re-) radiosurgery or re-irradiation [12]. Other novel treatment options for (recurrent) BM comprise e.g. Laser Interstitial Thermal Therapy (LITT) [13] or brachytherapy [14].

Surgery is well established as a first-line treatment for larger and symptomatic BM. However, the role of surgery for pretreated, recurrent BM is not yet defined, and only scarce data, originating from the pre-molecular era, are available. Only a few studies have reported on the feasibility of (re-)surgery in patients with single or multiple recurrent BM [8, 9, 15, 16]. They included narrowly defined patient cohorts previously treated by either surgery [8, 15] or sRS [16, 17], and reported median survival rates after resection of between 7.5 and 11.5 months. With 11.1 months the survival rate in the present study was within the range of the previously reported data. Furthermore, we did not analyze a narrowly defined cohort, but included patients with heterogenous primary tumors as well as a variety of administered prior treatments. The high rate of fatal cerebral progression in this series compared to previous studies may be due to the fact, that besides surgery, most therapeutic options had already been used, leading to a lack of salvage treatment in the case of further cerebral progression. As surgical resection may result in rapid symptom release by reducing the mass effect, the subsequent improvement in the patient’s clinical condition, possibly in combination with a re-evaluation of the tumor’s molecular status, may represent the major benefit of surgery. Since a fair clinical status is a prerequisite for radio-oncological and a tailored adjuvant treatment, this may positively influence the outcome, as observed before [18]. However, this benefit could not be observed with statistical significance for the patients in the present study.

Probably, the specific condition of this study’s population offers an explanation since it comprises patients who had already undergone extensive oncological treatment and a possible subsequent development of resistance mechanisms may leave few remaining therapeutic approaches in such patients.

In cases of extensive pretreatment by radiotherapy, resection might therefore be the only local treatment option left. As the cerebral progression partly reflects treatment failure of previous irradiation, the negligible impact of postoperative radiotherapeutic measures on either EFS or OS in this present study is not surprising.

The major argument for surgery in this patient cohort may be seen in the clinical improvement which is, in line with the current literature, the strongest predictor for further survival after recurrent BM treatment [4, 8, 17]. In this context less invasive local treatments such as LITT or brachytherapy may therefore not be suitable in situations with space-occupying lesions and/or symptomatic edema. As a consequence treatment results after resection in the present cohort may not be compared to other local treatment effects.

Also no treatment paradigm can be generated based on this present data due to an extremely heterogeneous population presenting with recurrent BM in clinical practice.

As opposed to the clinical improvement mentioned above, the postoperative complication rate was high and included a critical number of life-threatening complications. This is in contrast to other studies reporting on resection in the setting of initial BM diagnosis, where neurosurgery was usually well tolerated and proved to be feasible and safe [8, 15–17, 19, 20].

These results are the more surprising as all patients were treated at a specialized center and time of hospital stay was not different from other cranial surgical procedures. The high incidence of complications may therefore be mainly explained by the general condition of oncological patients. The underlying malignancy and/or multiple varied (systemic) pre-treatments may have impaired wound healing and hemostasis, and increased the risk for cardio-pulmonary complications [4, 21]. Furthermore, patient age was described as independently correlating with clinical outcome, since comorbidities are more common in elderly patients [4, 22, 23]. In this context, the indication for re-resection of BM must be based upon multidisciplinary consent that takes into account the patients´ general condition, the possible (and probable) clinical benefit, and the availability of further treatments.

## Conclusions

Surgical resection of recurrent BM may improve patients´ clinical status and possibly indirectly prolong survival but carries a high risk for surgery-related complications. Thus, careful patient selection in a multidisciplinary comprehensive treatment setting is mandatory, since a uniform treatment-paradigm cannot be established due to the heterogeneous patient cohort.

## Data Availability

The datasets used and/or analyzed during the current study are available from the corresponding author on reasonable request.

## Abbreviations

BM: brain metastases
BT: brachytherapy
CSF: cerebrospinal fluid
CTCAE: Common Terminology Criteria of Adverse Events
CTx: chemotherapy
EFS: event free survival
fRT: fractioned radiotherapy
GIT: gastro-intestinal tumor
HR: Hazard ratio
KPS: Karnofsky performance scale
LITT: Laser Interstitial Thermal Therapy
MRI: Magnetic resonance imaging
NCCN: National Comprehensive Cancer Network
NCI: National Cancer Institute
NSCLC: non-small cell lung cancer
OS: overall survival
PET: positron emission tomography
RCC: renal cell cancer
SCLC: small cell lung cancer
sRS: stereotactic radiosurgery
TT: targeted therapy
WBRT: whole brain radiation therapy

## References

[1] Shen CJ, Lim M, Kleinberg LR (2016). Controversies in the Therapy of Brain Metastases: Shifting Paradigms in an Era of Effective Systemic Therapy and Longer-Term Survivorship. Curr Treatm Opt Oncol.

[2] Steeg PS, Camphausen KA, Smith QR (2011). Brain metastases as preventive and therapeutic targets. Nat Rev Cancer..

[3] Chamberlain MC, Baik CS, Gadi VK, Bhatia S, Chow LQM (2017). Systemic therapy of brain metastases: Non-small cell lung cancer, breast cancer, and melanoma. Neurooncology.

[4] Sankey EW, Tsvankin V, Grabowski MM, Nayar G, Batich KA, Risman A (2019). Operative and peri-operative considerations in the management of brain metastasis. Cancer Med.

[5] Kalkanis SN, Kondziolka D, Gaspar LE, Burri SH, Asher AL, Cobbs CS (2010). The role of surgical resection in the management of newly diagnosed brain metastases: A systematic review and evidence-based clinical practice guideline. Jour Neuro-Oncol.

[6] Olson JJ, Kalkanis SN, Ryken TC (2019). Congress of Neurological Surgeons Systematic Review and Evidence-Based Guidelines for the Treatment of Adults with Metastatic Brain Tumors: Executive Summary. Clin Neurosurg.

[7] Ammirati M, Cobbs CS, Linskey ME, Paleologos NA, Ryken TC, Burri SH (2010). The role of retreatment in the management of recurrent/progressive brain metastases: A systematic review and evidence-based clinical practice guideline. Jour Neuro-Oncol.

[8] Schackert G, Schmiedel K, Lindner C, Leimert M, Kirsch M (2013). Surgery of recurrent brain metastases: retrospective analysis of 67 patients. Acta Neurochir.

[9] Kano H, Kondziolka D, Zorro O, Lobato-Polo J, Flickinger JC, Lunsford LD (2009). The results of resection after stereotactic radiosurgery for brain metastases: Clinical article. J Neurosurg.

[10] Atkinson TM, Ryan SJ, Bennett AV, Stover AM, Saracino RM, Rogak LJ (2016). The association between clinician-based common terminology criteria for adverse events (CTCAE) and patient-reported outcomes (PRO): a systematic review. Support Care Cancer.

[11] Kluetz PG, Chingos DT, Basch EM, Mitchell SA (2016). Patient-Reported Outcomes in Cancer Clinical Trials: Measuring Symptomatic Adverse Events With the National Cancer Institute’s Patient-Reported Outcomes Version of the Common Terminology Criteria for Adverse Events (PRO-CTCAE). Am Soc Clin Oncol Educ Book.

[12] Iorio-Morin C, Mercure-Cyr R, Figueiredo G, Touchette CJ, Masson-Côté L, Mathieu D (2019). Repeat stereotactic radiosurgery for the management of locally recurrent brain metastases. J Neurooncol.

[13] Chen C, Lee I, Tatsui C, Elder T, Sloan AE (2021). Laser interstitial thermotherapy (LITT) for the treatment of tumors of the brain and spine: a brief review. J Neurooncol.

[14] Ruge MI, Suchorska B, Maarouf M, Runge M, Treuer H, Voges J (2011). Stereotactic 125Iodine Brachytherapy for the Treatment of Singular Brain Metastases: Closing a Gap?. Neurosurgery.

[15] Bindal RK, Sawaya R, Leavens ME, Hess KR, Taylor SH (1995). Reoperation for recurrent metastatic brain tumors. Jour Neuro.

[16] Vecil GG, Suki D, Maldaun MVC, Lang FF, SaWaya R (2005). Resection of brain metastases previously treated with stereotactic radiosurgery. J Neurosurg.

[17] Kano H, Kondziolka D, Zorro O, Lobato-Polo J, Flickinger JC, Lunsford LD (2009). The results of resection after stereotactic radiosurgery for brain metastases: Clinical article. JNS.

[18] Schödel P, Jünger ST, Wittersheim M, Reinhardt HC, Schmidt N-O, Goldbrunner R (2020). Surgical resection of symptomatic brain metastases improves the clinical status and facilitates further treatment. Cancer Med.

[19] Kamp MA, Dibué M, Niemann L, Reichelt DC, Felsberg J, Steiger H-J (2012). Proof of principle: supramarginal resection of cerebral metastases in eloquent brain areas. Acta Neurochir (Wien).

[20] Kamp MA, Dibué M, Santacroce A, Zella SM, Niemann L, Steiger H-J (2013). The tumour is not enough or is it? Problems and new concepts in the surgery of cerebral metastases. Ecancermedicalscience.

[21] Gaspar L, Scott C, Rotman M, Asbell S, Phillips T, Wasserman T (1997). Recursive partitioning analysis (RPA) of prognostic factors in three Radiation Therapy Oncology Group (RTOG) brain metastases trials. Int J Radiat Oncol Biol Phys.

[22] Moazami N, Rice TW, Rybicki LA, Adelstein DJ, Murthy SC, DeCamp MM (2002). Stage III non–small cell lung cancer and metachronous brain metastases. J Thorac Cardiovasc Surg.

[23] Stark AM, Tscheslog H, Buhl R, Held-Feindt J, Mehdorn HM (2005). Surgical treatment for brain metastases: prognostic factors and survival in 177 patients. Neurosurg Rev.

